# Design and preliminary implementation of onsite electrochemical wastewater treatment and recycling toilets for the developing world†

**DOI:** 10.1039/c8ew00209f

**Published:** 2018-08-07

**Authors:** Clément A. Cid, Yan Qu, Michael R. Hoffmann

**Affiliations:** a Linde-Robinson Laboratories, California Institute of Technology, 1200 E California Blvd, Pasadena, CA 91125, USA; b Trussell Technologies Incorporation, 232 N Lake Ave., Suite 300, Pasadena, California, 91101, USA

## Abstract

Self-contained toilet wastewater treatment system prototypes based on electrochemical oxidation of feces and urine using bi-layered semiconductor anodes ([Bi_2_O_3_]*_z_*[TiO_2_]_1−z_/Ir*_x_*Ta*_y_*O_2_/Ti) have been designed, constructed, and implemented in regions where access to proper and sufficient sanitation is limited. Prototypes were designed to fit in shipping containers in order to provide toilets and onsite wastewater treatment with clean water recycling. Units were designed to handle the waste of 25 users per day (or 130 L of toilet wastewater). The first prototype was tested on the Caltech campus (Pasadena, California) followed by improved second-generation prototypes that were subsequently installed in India (Ahmedabad, Gujarat and Kottayam, Kerala) and China (Yixing, Jiangsu) for open use in various public settings. The prototypes were able to provide for the disinfection of pathogens (<10 MPN *Total coliforms* and <1 MPN *Fecal coliform* indicator organisms per 100 mL), reduction of chemical oxygen demand (<100 mg O2 L−1), ammonia (<10 mg N L−1), and color at an average energy consumption of less than 180 W h per user per day. The treated wastewater was recycled for use as toilet flushing water.

Water impact

More than 2.6 billion people do not have access tosanitation. With an average energy usage of less than 180 W h per user per day, electrochemical toilet wastewater disinfection, chemical oxygen demand removal, and ammonia removal can provide a viable and cost-effective alternative to traditional sewers. Onsite electrochemical wastewater treatment systems were tested and performed without critical breakdown during the field testing.

## 1 Introduction

**Fig. 1:**
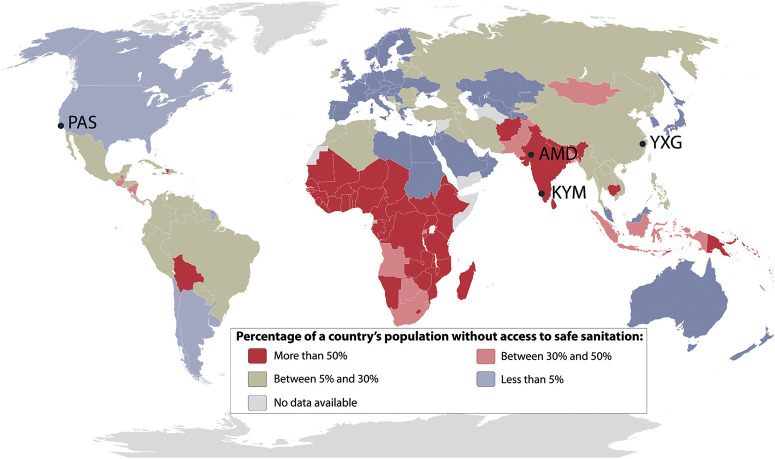
Percentage of a country's population without access to safe sanitation in 2015 according to the World Health Organization.^58^ Location of the four prototype testing sites across the world: PAS, Pasadena, California, USA; AMD, Ahmedabad, Gujarat, India; KYM, Kottayam, Kerala, India; YXG, Yixing, Jiangsu, China.

In February 2011, The Bill & Melinda Gates Foundation (BMGF) announced a major challenge to university researchers to “Reinvent the Toilet”. The primary goal of the BMGF was to engage universities in the development of new and innovative methods to treat human bodily wastes at the site of origin without discharge to the ambient environment or discharge to conventional sewer systems, septic tanks, cesspools, or open drainage systems. The overarching goal of the BMGF Global Development Program within the context of their Water, Hygiene and Sanitation initiative was to develop practical low-cost solutions that could be implemented in regions of the world that lack access to safe and affordable sanitation. The primary challenge was to develop a comprehensive approach to design, development, testing, and prototyping of systems that could collect and process human waste on-site at the source of origin and at the same time produce useful byproducts including fertilizer, mineral salts, energy, purified, and disinfected water with no solid or liquid discharge to the environment. The overarching objective is to provide suitable sanitary systems for the 2.6 billion people who currently lack access to safe and affordable sanitation (Fig. 1). A cost constraint was set at a maximum of $0.05 per person per day include capital costs and operating expenses.

The development of integrated networks and facilities for the transport and subsequent treatment of domestic wastewater has been a key factor in the growth and development of modern urban environments. Sanitation has accompanied human development from early civilizations with rudimentary systems^1^ to mid-19th century first large-scale sewer networks in American and European cities.^2–4^ Although a well-constructed modern urban sewer network can be hygienic and efficient due to economies of scale,^5^ they also have major drawbacks, which include nuisance odors from improper operation and maintenance, ^6^ local groundwater contamination due to leakage from improper connections and corrosion of pipes and concrete sewers,^7,8^ or prohibitively expensive capital investments.^9^

For these reasons, developing countries have often turned to non-sewered sanitation (NSS) systems for the disposal of human bodily waste. Furthermore, in areas with limited access to water, technologies have traditionally been restricted to dry or manual pour-flush types of toilets such as composting toilets or pit latrines.^10^ Although these waterless technologies appear attractive because of their limited need for water, they do not provide the olfactory or sanitary comfort of flush toilets.^11^ In addition, they are not always reliable for disinfection, pathogen removal,^12^ or for preventing subsequent pollution by latrine waste soils and groundwater.^13^ In areas that have access to water, decentralized toilets using flush technologies are most often connected to septic tanks. Septic tank treatment systems require large land surface areas to build effective leaching fields that are necessary for the safe elimination of pathogens (Title V septic system in the United States) and can often lead to fecal contamination of local water sources if improperly installed and maintained.^14–16^

Therefore, a technology capable of treating and recycling toilet wastewater at low cost would have significant advantages over traditional NSS solutions (*vide supra*).In this regard, Radjenovic and Sedlak have identified electrolysis processes such as potentiostatic electrochemical oxidation as “potential nextgeneration technologies for the treatment of contaminated water”. ^17^ Electrochemical oxidation of wastewater has been investigated for more than 30 years with a focus on organic pollutant degradation,^18,19^ most systems rely on the anodic formation of free hydroxyl radical OH˙ from water oxidation or the direct oxidation of the compounds of interest. Both processes consume a lot of energy to achieve appropriate contaminant removal.^20^

Weres and collaborators^21–23^ investigated the use of multilayer semiconductor anodes to generate surface-bound hydroxyl radicals OH˙ for organics degradation.^24–26^ These multilayer semiconductor anodes have a low overpotential for the oxidation of chloride to chlorine.^27^ This capability makes the multi-layer semiconductor anodes particularly suitable for the direct formation of reactive chlorine species (RCS) from the oxidation of the chloride naturally present in the human wastewater.^28–31^ Although the previously published laboratory results have shown the feasibility of anodic oxidation for toilet wastewater treatment,^29–32^ there is no literature available about the automated, autonomous on-site electrochemical treatment of toilet wastewater under actual field operating and testing conditions. Herein, we present the results of field studies employing electrochemical wastewater treatment for the removal of chemical oxygen demand and for recycling of disinfected and clarified water for use as toilet flushing water.

## 2 Guidelines, materials and methods

### 2.1 Health considerations for an onsite wastewater recycling systems

The primary sources of biological and chemical contamination entering onsite wastewater treatment systems are from human excreta. The amount and the composition of human excreta varies greatly from one individual to another^33,34^ with an average of 1 L to 1.5 L of urine and 300 g to 450 g of feces per adult per day. Feces are often the major carrier of pathogens in human excreta^35^ with an average number of 10^11^ CFU (colony-forming units) of bacteria per gram of feces for a healthy adult individual. Given that pathogen die-off times in untreated human excreta are between one and three months for bacteria and viruses, and several months for helminth eggs,^36^ a reliable and rapid removal of pathogens down to acceptable levels is crucial for the success of an onsite human wastewater treatment technology. For example, the World Health Organization considers that a safe pathogen level appropriate for water reuse in agriculture is less than 1 CFU per 100 mL for typical indicator organisms *E. coli*.^37^

When onsite wastewater treatment systems are installed close to their users in order to minimize installation costs, such systems can become potential threats to the health of humans living nearby when the wastes are not properly contained^38^ or sufficiently treated. Natural barriers such as the leaching fields for septic tanks or clay or concrete walls for dry latrine pits are not always effective barriers.^13,39^ Risks of contamination are further increased when the users come into contact with effluent streams (*e.g*., treated water and/or biosolids) produced by onsite sanitation systems. Thus, the treated and recycled waters that are processed onsite must be free of pathogens and have an acceptable physicochemical composition that meets conventional water quality standards for reuse.

However, technical standards that have been adopted in many countries help to regulate the composition of recycled water for domestic reuse but they are often limited to large scale indirect and direct potable reuse of conventional wastewater treatment plant effluents^40^ or the treatment and reuse of non-fecal contaminated water (greywater) primarily from sinks, washers, and showers (NSF/ANSI Standard 350). Therefore, a toilet wastewater recycling system has to produce an effluent that does not damage the system itself, is safe for users, and contains enough residual disinfecting capacity to prevent subsequent chemical and microbial contamination due to exposure to the treated and recycled water.

In addition to the meeting the basic sanitary and water quality requirements, a self-contained toilet and wastewater treatment system for use in developing countries needs to be affordable, durable, and functional in an off-grid environment with limited access to electricity, fresh water, and sewers.

### 2.2 Choice of prototype testing locations

**Fig. 2:**
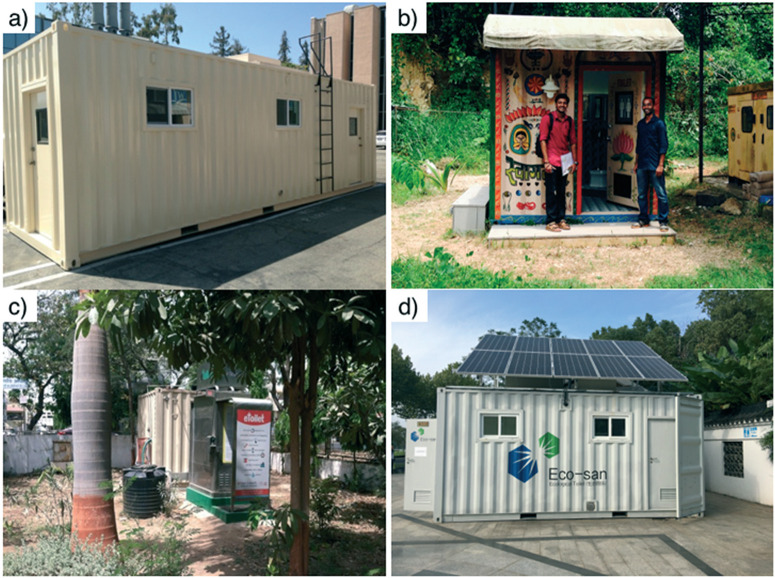
Caltech solar toilet system prototypes: a) prototype PAS (Pasadena, CA); b) prototype KYM (Kottayam, Kerala, India); c) prototype AMD (Ahmedabad, Gujurat, India); d) prototype YXG (Yixing, Jiangsu, China).

Four pre-alpha prototypes of a similar design (Fig. 2 and section 3) were tested in the USA, India, and China (Fig. 1). All the testing sites had a connection to sewers in case the prototypes would fail during testing. The first pre-alpha prototype was tested on the campus of the California Institute of Technology (Caltech) in Pasadena, California (PAS prototype, Fig. 2a) for preliminary data gathering and early design adjustments; two additional pre-alpha prototypes (AMD and KYM) were designed and built on the Caltech campus and then shipped to India for 6 month field trials in two different locations. The quantity of treated wastewater was recorded for each prototype and the frequency of usage of the toilet was recorded for the prototype designated as AMD only. All prototypes were designed to be running full time. The prototype designated as AMD (Fig. 2c) was installed in a public park in the city of Ahmedabad in Gujarat State of northwest India. Ahmedabad has a semi-arid climate and is the sixth largest city of India with more than 6.3 million inhabitants. The prototype designated as KYM (Fig. 2b) was installed in the campus of Mahatma Gandhi University near the School of Environmental Sciences in Amalagiri district of Kottayam City in the State of Kerala, which is located in southwestern India. Kottayam has a tropical climate with a population of 200 000 inhabitants. A fourth pre-alpha prototype was constructed and tested in the Municipal Yixing Elementary School of Yixing, China (YXG prototype, Fig. 2d) in collaboration with Yixing Eco-Sanitary Manufacture Co.

### 2.3 Monitoring and evaluation methods

Chemical oxygen demand,^30^ total nitrogen,^41^ total suspended solids (TSS), and indicator organisms *E. coli, Total coliform, and Fecal coliform* bacteria were measured to assess the wastewater treatment efficacy.

The voltage at the electrodes and the current delivered by the power supply to the electrode arrays were continuously measured using a personalized data logger (Programmed Scientific Instruments, Arcadia CA) at regular intervals (*e.g*., every 10 seconds). The activation and deactivation of pumps as well as the status of water level sensors were monitored and recorded using the same data logger. At each recording time, the data logger stored a new line of values of the different components of the system in a daily comma separated values (CSV) file. The data was tagged with the local time and date and stored in a solid-state device in the computer controlling the system. The CSV files were regularly retrieved by an operator.

### 2.4 Analytical methods

Unfiltered COD was measured using a reflux digestion system with water condensers followed by titration according to Standard Method 5220 (ref. 42) or *via* colorimetric method similar to Hach Method 8000 (Hach Company, Loveland CO). TN was determined using persulfate digestion (Hach Method 10 071). Total Kjeldahl Nitrogen (TKN) was determined by distillation (Indian Standard 5194-1969). Cl^−^, NH_4_^+^ + NH_3_, Ca^2+^, and Mg^2+^ concentrations were determined by ion chromatography (Dionex ICS 2000; AS19G anions, CS12A cations).

Disinfection was assessed by estimating the quantity of indicator organisms *E. coli, Total coliforms, and Fecal coliforms* with the following respective EPA methods: 1103.1,^43^ 9132,^44^ and 1680 (ref. 45) with appropriate dilutions. Free chlorine (FC) was measured by reaction with *N,N*-diethyl-*p*-phenylenediamine (DPD) indicator in accordance with Standard Method 4500-Cl G^42^ and Hach Method 8021. Total chlorine (TC) was measured by the Amperometric Titration Method in accordance with Standard Method 408 C.^42^

Cathodic and anodic potentials relative to normal hydrogen electrode (*vs*. NHE) were measured using a 3.5 M silver/ silver chloride (Ag/AgCl, E*_0_* = 0.205 V *vs*. NHE) reference electrode (RE-5B, Bioanalytical Systems Inc., USA) connected to a three-electrode potentiostat (Biologic, France) measuring the potential between the reference electrode and the anode used for chlorine production (see below).

## 3 Design of the self-contained toilet and treatment systems

### 3.1 Sizing considerations

**Fig. 3:**
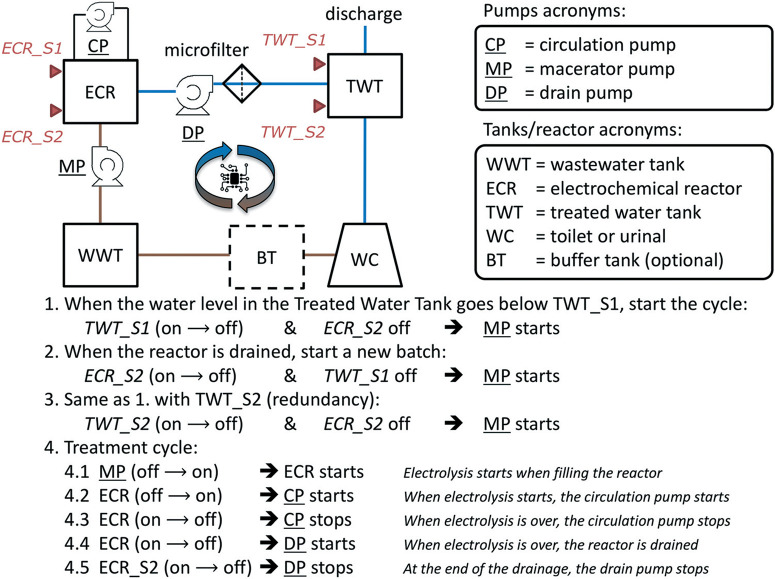
System flow diagram (top left, see Fig. S1† for volumes and residence times) with automation algorithm description for the onsite toilet wastewater treatment and recycling systems. Pumps are underlined. Capacitive level sensors are represented by red triangles. Brown lines illustrate the flow of untreated wastewater while blue lines illustrate the flow of treated and recycled wastewater.

**Fig. 4:**
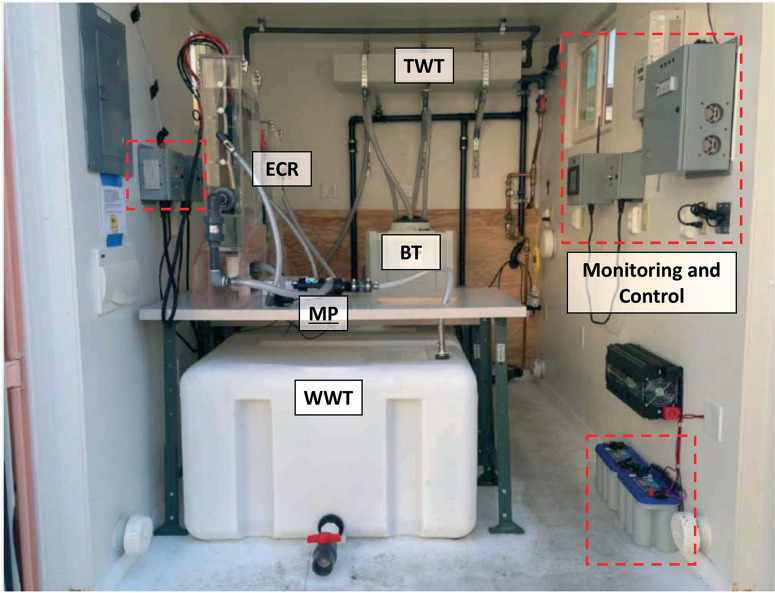
Photograph of the layout of one of the self-contained electrochemical treatment prototypes installed in the field. The combined power, monitoring, and control system is highlighted in red dashes. Refer to Fig. 3 for definition of the acronyms.

A flow diagram of the overall toilet wastewater treatment process is presented Fig. 3 and S1† and a picture of a typical treatment system as installed in the field-tested prototypes (PAS, AMD, KYM) is reproduced Fig. 4. When the flush toilet was used, the mix of urine, feces, and flush water (toilet wastewater) was macerated and pumped (Jabsco Macerator pump 18590-2094, Xylem USA or Saniflo Sanigrind Grinder pump for Bottom Outlet Toilets, SFA France) into a 2 m^3^ polypropylene sedimentation tank for a residence time of τ_bio_ ≥ 15 days. The maceration reduced the feces to particles of 3 mm or less according to the manufacturer's description. In the sedimentation tank, the toilet wastewater underwent decantation and some level of anaerobic digestion, similarly to a septic tank.^46^ The decanted solids remained at the bottom of the sedimentation tank without further active processing. The prototypes were designed for treating the toilet waste from approximately 25 daily uses: this is the equivalent to having a single toilet for a family of five people, the average household size in India in 2011.^47^ Each of the family members flushing five times per day on average^48^ with a flush volume of 1.28 US gallons or 5 L (US EPA WaterSense) and considering that one person produces approximately 1.5 L of urine in one day,^41^ the total daily volume of toilet wastewater to treat was then estimated to be *V*_d_ = 132 L per day so the sedimentation tank should be sized to hold at least τ_bio_ × *V*d ≈ 2 m^3^.

After decanting in the sedimentation tank, the wastewater was macerated and pumped (Jabsco Macerator Pump 18590, Xylem USA) to an electrochemical reactor (ECR) system (see below for description) for batch processing at constant voltage with active recirculation (10 L min^−1^) during a period τ_elec_, the electrochemical residence time. The working volume needed for the ECR *V*_ECR_ was determined by (eqn (1)) with α the fraction of the daily time during which the ECR is running.

$$V_{\text{ECR}}\;=\;\frac{V_{\text{d}}}{24\alpha \tau_{\text{elec}}}\;\left(\left(=\;22\;\text{L}\right)\right)$$( 1 )

After electrolysis in the ECR for a period τelec, the water was filtered through a 200 μm-mesh microfiltration unit (Grainger USA). The treated effluent was then pumped with a drain pump (Jabsco PAR Max 3, Xylem USA) to a storage tank to use for flushing the toilet. The treated water tank (TWT) was capable of storing flushing water for one day of operation. An overflow mechanism was installed in the TWT in case excess treated water needed to be discharged from the system occasionally. The sizing of the polypropylene sedimentation tank as well as the water losses through evaporation during the process significantly limited the necessity to discharge treated water. Four complete toilet and associated wastewater treatment and recycling systems were operated in the US, China, and India (Table 1).

**Table 1:** Information about the different toilet wastewater treatment and recycling units installed in the world

Configuration	Ref	Location	Testing period	Average daily usage during testing
Self-contained bathroom + wastewater treatment and recycling unit in a shipping container	PAS	Pasadena, USA	06/2013 to 06/2016	<5
	KYM	Kottayam, India	04/2014 to 01/2016	6
	YXG	Yixing, China	12/2014 to 05/2015	35
Wastewater treatment and recycling unit connected to an “eToilet” public toilet (Eram Scientific, Trivandrum, Kerala, India)	AMD	Ahmedabad, India	04/2014 to 01/2016	7

### 3.2 Electrochemical reactor system

The ECR tank body was made of a polyĲmethyl methacrylate) (PMMA) welded together (Nanopac. Yongin-Gun, South Korea) in a rectangular cuboid shape with the following dimensions: 63.5 cm × 35.6 cm × 16.5 cm (height × width × depth; Fig. S2†). Two 6 mm thick PMMA plates of respective dimensions 25 cm × 14 cm and 56 cm × 14 cm with 1.5 cm diameter holes spaced every 2.5 cm were used to hold the electrode array in place at a distance of 7 cm above the bottom of the ECR. 0.75 inch and 1 inch diameter National Pipe Tapered (NPT) thread holes were drilled on the side and the bottom of the ECR tank to connect sampling ports and plumbing. Circulation of fluid inside the ECR tank was assured by a brushless centrifugal pump (Fortric ZKWP04 24V, Fortric China). The ECR tank was connected to the other components of the system using braid-reinforced polyurethane hose or polyvinyl chloride (PVC) pipes of sufficient diameter.

At the core of the ECR, electrode plates were assembled as an array in alternate configurations of doubly-coated anodes sandwiched between two stainless-steel cathodes (*e.g*., CA*_n_*CA*_n_*_+1_…C, etc.) where each electrode plate was separated by a 3 mm spacing; nylon screws, nuts, and washers were used for structural integrity. The arrays composed of eight stainlesssteel (316 Grade) cathodes “C” and seven doubly-layered semiconductor anodes “A” ([Bi_2_O_3_]*_z_*[TiO_2_]_1−_*_z_*/Ir_x_Ta*_y_*O_2_/Ti) (Nanopac, South Korea) that were coated on both sides. The manufacturing process and the effect of the outer layer composition ([Bi_2_O_3_]*_z_*[TiO_2_]_1−_*_z_*) have previously been described in the literature. ^28,31^ The total exposed surface area of the anodes (*S*_A_) was 1.8 m^2^. The ECR electrode array was powered at an electrical potential between 3.3 V and 3.5 V using a potentiostatic power supply (Program Scientific Instruments, USA).

### 3.3 Automation for the wastewater treatment and recycling

The daily number of users and the frequency of usage of the toilets were not controlled in any of the systems. For this reason, it was necessary to ensure that a sufficient amount of treated water was available for flushing at all time to support the continuous operation of the treatment system without direct supervision. This was achieved with a computercontrolled automation algorithm (Fig. 3) programmed on a dedicated software package (Program Scientific Instruments, Arcadia CA) running on a Panel PC PPC-L62T (Advantech, China) with Windows 7 operating system (Microsoft, USA). Capacitive level sensors CD50 DC (Carlo Gavazzi, Italy) were used as triggers for the automation mechanism. Pumps with a programmed maximum running time were used as actions (*e.g.*, macerator pump turns on) or as triggers when they changed state (*e.g*., circulation pump stops running). ECR power status (or change of status) was used as a trigger, an action, and a feedback for the automation algorithm.

The algorithm is composed of two main parts (Fig. 3): the start of a treatment cycle (lines 1–3) and the treatment cycle loop (lines 4.1–4.5). A treatment cycle starts when the level of water in the treated water tank falls below the sensor TWT_S1 (line 1), placed approximately at 20% of the tank's height. The macerator pump MP switches on for a fixed duration to fill up the ECR tank, the ECR power supply starts, and the circulation pump CP starts. At the end of the treatment cycle (τ_elec_), the ECR tank is emptied by the drain pump DP into the treated water tank TWT. After that, if the water level in TWT is still below TWT_S1, a new treatment cycle begins. The treatment cycles will continue until the level of the treated water tank is above the TWT_S2 sensor, positioned close to 90% of the tank's height.

### 3.4 Energy distribution across to the system

The energy consumption of the entire system and ECR was monitored using non-invasive current sensors installed on the wires connecting the control system to its power source. The power source was a combination of grid electricity at 220–240 V AC when available, and 24 V DC power source from a 330 W solar panel (Xunlight, USA) stored in two 12 V Blue Top lead-acid backup batteries (Optima, USA) via a Conext MPPT 60 PV (Schneider Electric, Germany) charge controller (Fig. S3†). A backup battery recharge was also implemented using a TRUECharge 2 40 A (Xantrex, USA) battery charger connected to grid electricity.

### 3.5 Integration

All components of the entire system were housed in customized steel shipping containers with an integrated public bathroom when necessary (Fig. S4†). AMD and PAS prototypes were modified 10 ft and 30 ft long containers cut from standard length 20 ft and 40 ft international shipping containers, respectively. KYM and YXG prototypes were repurposed 20 ft standard shipping containers. All containers were insulated and retrofitted with in-wall electrical wiring and on-wall plumbing in copper, cross-linked polyethylene (PEX), or PVC pipes. Doors and windows were added to improve access to the treatment system, increase air circulation, and provide a physical work environment for sampling and on-site measurements (Fig. 4).

## 4 Results and discussions

### 4.1 Free chlorine production

Chlorination is a cost-effective way to remove pathogens in water, if allowed sufficient contact time for a given free or total chlorine concentration.^49,50^ In our prototype systems, chlorine is continuously generated *via* the electrochemical oxidation of chloride, (*i.e*., the chlorine evolution reaction, CER), at a fixed potential 3.5 V ± 0.25 V across the electrodes. The CER is an apparent first-order reaction with respect to the concentration of chloride in solution. In a large-electrode array (ECR) as shown in (Fig. S5†), the CER rate is shown to vary quasi-linearly from 11 to 17 ppm Cl_2_ min^−1^ with respect to the ratio *S*A/*V*_ECR_ in 20 mM NaCl in water. After an extended period of operation of the system with wastewater, the CER rate in tested in 20 mM NaCl in water stabilized at a near constant level (Fig. S6†).

Due to a consistent input of urine into the system, the [Cl−] was found to variable at any point in time, in part, because the treated water that was recycled also allowed for a build-up of total chloride in the anaerobic holding tank. Variations in [Cl^−^] were observed depending on the type and frequency of usage as well as the location of the unit (Table 2). In theory, when the system is running at full capacity after an initial set up period, the steady-state [Cl^−^]_ss_ should be approximately equal to the concentration of chloride in urine, which ranges between 53 mM to 240 mM.^41^ However, lower concentrations (typically 10–20 mM) were actually observed (Table 2). The lower concentrations were most likely due to the following factors: the treated water tank was periodically loaded with tap water at the beginning of the testing period until the wastewater in the sedimentation tank reached at least 40% of volume and also because of the use of excess non-recycled water for additional flushing or for personal hygiene in the public bathrooms.

**Table 2:** Average toilet wastewater composition in the different prototypes. Ranges are given when available

Parameter	Unit	Prototype reference			
		PAS*^a^*	AMD*^b^*	KYM*^c^*	YXG*^c^*
COD	mg O_2_ L^−1^	150–250	100	335	550
Cl^−^	mmol L^−1^	11–20	11	15	24
NH_3_ + NH_4_^+^	mg NH_3_ L^−1^	80	30–40	235	480
PO_4_^3−^ + HPO_4_^2-^	mmol L^−1^	0.64		—	—
Alkalinity as CaCO_3_	mmol L^−1^	17	—	10.7	27
pH	—	8.3	7.4	7.5	8.5

a After 16 months of collection and 6 months of recycling water.

b No recycled water used.

c After 2 months of running.

For an electrical potential of 3.5 V between the anodes and cathodes, the measured anodic potential is 1.4 ± 0.2 V *vs*. NHE and the cathodic potential is −2.1 ± 0.2 V *vs*. NHE. The cathodic potential is sufficient for the reduction of protons to hydrogen as it was previously described in the literature. This potential is sufficient for the production of surfacebound hydroxyl radicals on titanium dioxide at pH between 6 and 9 (ref. 51 and 52) but is not sufficient to generate free hydroxyl radicals (*E*_0_(HO˙) = 2.31 V *vs*. NHE) in solution.^53^

**Fig. 5:**
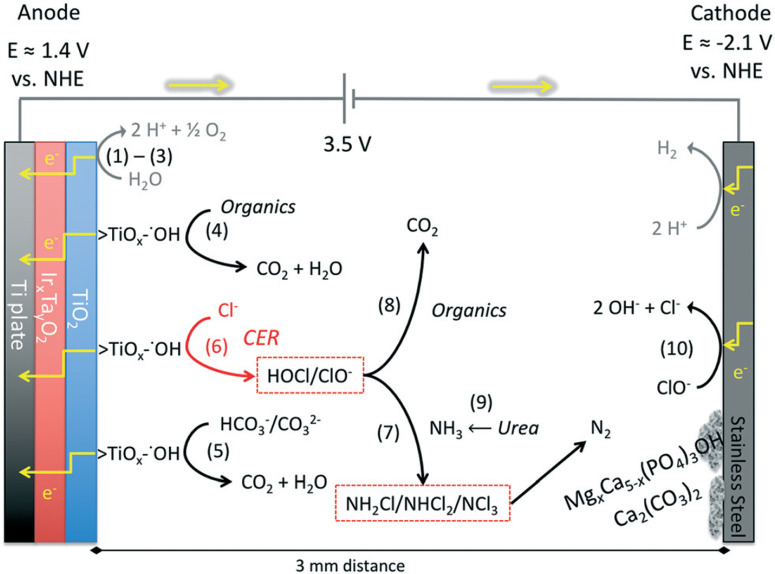
Electrons flow and main chemical reactions in the electrochemical reactor. (1)–(3) illustrate the oxidation of water on the TiO2-semiconductor oxide surface. (5) represents the direct oxidation of bicarbonates to CO2. (4) and (8) illustrate the removal of organics by direct (4) or indirect (8) oxidation. (6), (7), (8), and (10) illustrate the production and the fate of free chlorine (HOCl/ClO^−^) during electrochemical treatment. The yellow arrows represent the flow of electrons in the electrodes and across the wires.

The detailed electrochemical surface reactions (Fig. 5(1) through (6)), previously identified and classified by Comninellis^18^ consist of a two-step electron transfer for the oxidation of water on the surface of the metal-oxide electrode. In the case of TiO_2_, a one-electron surface oxidation of water locally reduces titanium from TiĲIV) to TiĲIII) with chemisorption of ˙OH (1); then the surface metal hydroxy adduct undergoes deprotonation with the concomitant release of an electron (2) and the corresponding oxidation of TiĲIII) back to TiĲIV) coupled with the formation of O_2_ or the direct surface oxidation of organic matter (3). Comninellis has also shown that the metalhydroxyl bond (>TiĲIII)OH˙) can directly oxidize electrondonating organic matter, leading to subsequent mineralization or simply oxidize TiĲIII) back to TiĲIV) with deprotonation and liberation of O_2_ (4). In the presence of chloride and a sufficient concentration, the surface-bound hydroxyl radical of >TiĲIII)OH˙ can directly oxidize Cl^−^ to form Cl_2_ and subsequently HOCl due to hydrolysis of molecular chlorine (6).^27^ FC is defined as the sum of concentrations of hypochlorous acid, [HOCl], and hypochlorite ion, [ClO^−^], which are in an acid–base equilibrium (HOCl ⇌ H^+^ + ClO^−^, p*K*_a_ = 7.53).

The measured CER rate in 20 mM NaCl solutions varied from 11 ± 0.5 ppm Cl_2_ min^−1^ before the electrodes had any contact with wastewater and decreased to 7 ± 0.7 ppm Cl_2_ min^−1^ after approximately 50 hours of electrolysis of toilet wastewater (Fig. S6†). The observed decrease in the rate of the CER was most likely due to the formation of a layer of organic compounds on the surface of the anodes. The net effect was a reduction in the CER rate by almost 40%; however, after stabilization of the CER, the removal of organic matter was stable (*vide infra*).

### 4.2 Removal of undesired organic and inorganic contaminants

**Fig. 6:**
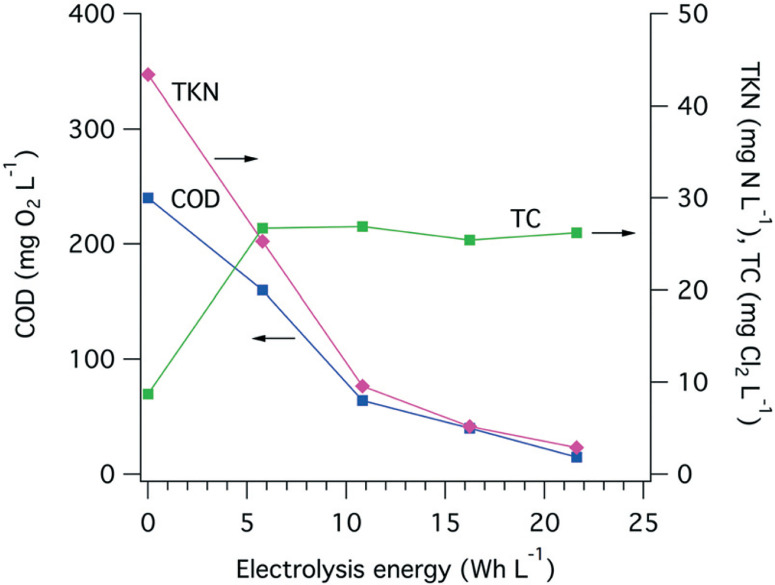
Evolution of the COD, TKN, and TC during the treatment of toilet wastewater in a single electrochemical treatment cycle in AMD prototype on 01/12/2015. Each point represent the average of three triplicate measurements.

**Fig. 7:**
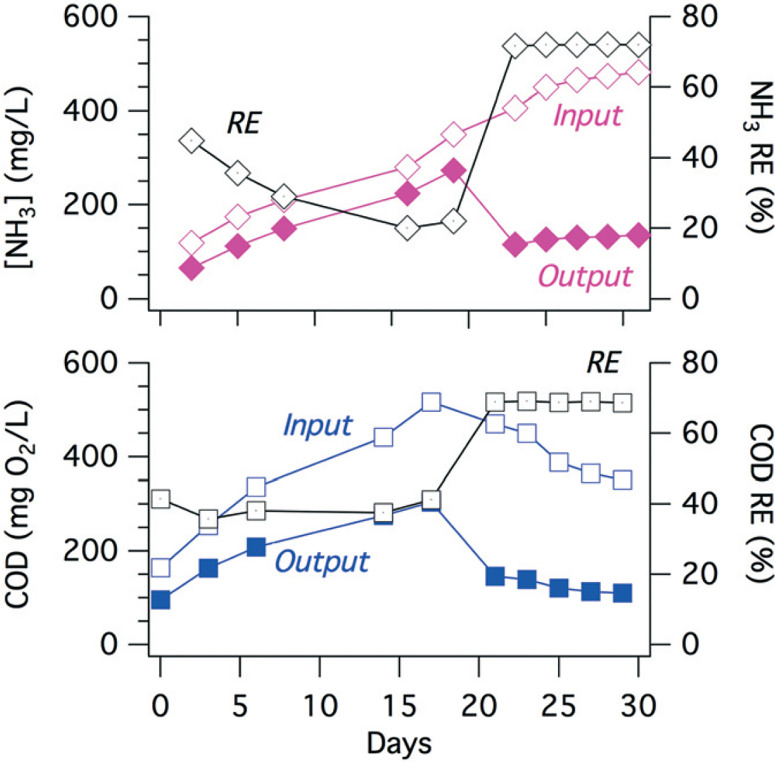
NH_3_ (top) and COD (bottom) averaged concentrations before (input) and after (output) an electrochemical treatment cycle of 4 hours with respective removal efficiencies (RE) for 30 continuous days of operation of YXG prototype. Day 0 corresponds to the beginning of usage of the prototype.

The electrochemically produced FC (Fig. 5(6)) can oxidize ammonia to form chloramines (7) while also oxidizing organic matter (8) present in the wastewater.^54^ The ammonia present in the collected toilet wastewater was formed primarily from the hydrolysis of urea (9). Although bicarbonate formed from the hydrolysis of urea combined with that generated via the oxidation of organic matter could interfere with the CER by adsorption on active anodic surfaces (5), thereby limiting the sites available for the oxidation of Cl− to FC. The increase in TC concentration during an electrolysis batch was correlated with the removal of TKN because formation of chloramines (Fig. 6). The TC concentration reached a steady state due to the reduction of chloramines to N_2_ in the solution as well as the reduction of ClO^−^ back to Cl^−^ at the cathode surfaces (10). For instance, the ammonia monitored in the YXG prototype over the first 30 days of operation (Fig. 7) showed 70% of NH_3_ removal efficiency once the system stabilized. The stabilization period was probably due to the fact that the system was started with significant amount of non-recycled water in the wastewater tank. This led to a high dilution of urine and feces for the first 15 days of operation.

The oxidation and mineralization of the organic matter (Fig. 5(4) and (8)) was observed through the decrease of COD during electrolysis (Fig. 6). For instance, the COD monitored in the AMD prototype over 1000 hours of operation showed between 70% and 80% removal efficiency when the chloride concentration was above 500 ppm and the applied potential was 3.5 V (Fig. S9† and Table 4). Furthermore, COD removal kinetics from prototype KYM (Fig. S7†) were consistent with the first-order kinetic model expressed by Martinez-Huitle and Ferro51 for transport-limited electrolytic oxidation with fitting coefficients reproduced Table S1.†

### 4.3 Disinfection

A summary of disinfection analysis performed at the KYM unit installed in Kottayam, India is reproduced Table 3: disinfection occurred after 2 to 3 hours of treatment (equivalent to 10–20 W h L^−1^ electrolysis energy), as indicated by the levels of the major indicator organisms being below the detection limit (*Fecal coliforms and E. coli*) or below drinking water safety standards (*Total coliforms*). These results are in accordance with the amount of chlorine and chloramines produced as well as the residence time in the ECR: 25 ppm TC assumed to be mostly chloramines because breakpoint chlorination was not reached; the equivalent contact time Ct value for 4 hours operation was greater than 6000 mg min L^−1^, which is more than 5 times higher than the recommended Ct value for 3-log inactivation of *Giardia* cyst at 20 °C, and almost 10 times of recommended Ct value for 4-logs virus inactivation at 20 °C.^55^ Similar results were observed in AMD and PAS units.

**Table 3:** Indicator organisms *Total coliform, Fecal coliform*, and *E. coli* detection test results during electrochemical treatment cycles. Analysis performed by the Topical Institute of Ecological Sciences of Mahatmah Gandhi University (Kottayam, Kerala, India) and Albio Technologies (Kochi, Kerala, India)

Reaction time (energy consumed)	*Total coliforms*	*Fecal coliforms*	*E. coli*
	MPN/100 ml	MPN/100 ml	CFU ml^−1^
11/17/14			
0 h (0 W h L^−1^)	>1100	>1100	200
2 h (11 W h L^−1^)	<1	<1	<1
4 h (22 W h L^−1^)	<1	<1	<1
3/28/15			
0 h (0 W h L^−1^)	>2400	75	Present
1 h (4.1 W h L^−1^)	1100	0	Absent
2 h (8.2 W h L^−1^)	23	0	Absent
3 h (12 W h L^−1^)	15	0	Absent
7/25/15			
0 h (0 W h L^−1^)	>2400	120	Present
1 h (6.7 W h L^−1^)	1100	75	Present
2 h (13 W h L^−1^)	93	4	Present
3 h (20 W h L^−1^)	43	3	Present
4 h (27 W h L^−1^)	9	0	Absent
9/18/15			
0h(0 WhL^−1^)	75	0	Absent
2 h (12 W h L^−1^)	23	0	Absent
4 h (24 W h L^−1^)	9	0	Absent

**Table 4:** Typical wastewater quality parameters measured before and after a 3 hour electrolysis cycle over the course of the field testing of the AMD prototype. Values are average of three replicates

Date(dd/mm/yyyy)	Unfiltered COD (mg O2 L−1)		TKN or NH_3_ (mg N L^−1^)		TSS (mg L^−1^)		Chloride (mg Cl^−^ L^−1^)		Total/free chlorine (mg Cl_2_ L^−1^)	
	Before	After	Before	After	Before	After	Before	After	Before	After
18/09/2014	32	—	n.d.	—	—	—	—	—	—	—
14/10/2014	100	—	31	—	100	—	—	—	—	—
03/11/2014	90	n.d.	—	—	100	50	182	35.1	—	—
29/11/2014*^a^*	43	11	15	2	—	—	100	30	—	—
23/02/2015	100	48	—	—	—	—	—	—	—	—
27/08/2015	—	—	—	—	—	—	—	—	0*^b^*	39*^b^*
01/12/2015	240	40	43	5	—	—	—	—	—	—
16/01/2016	223	26	—	—	—	—	235	203	<1*^c^*	—
02/02/2016	371	95	—	—	—	—	886	382	<1*^c^*	2.73*^c^*
31/03/2016	320	50	1.05*^d^*	2.04*^d^*	245	87	—	—	—	—
11/04/2016	234	56	25*^d^*	40*^d^*	180	35	425	390	<1*^c^*	<1*^c^*

a Analyses performed by third-party (Ahmedabad Municipal Corporation Central Lab). Total nitrogen was measured.

b Total chlorine.

c Free chlorine.

d Potential interference for ammonia measurement.

### 4.4 Energy consumption

**Fig. 8:**
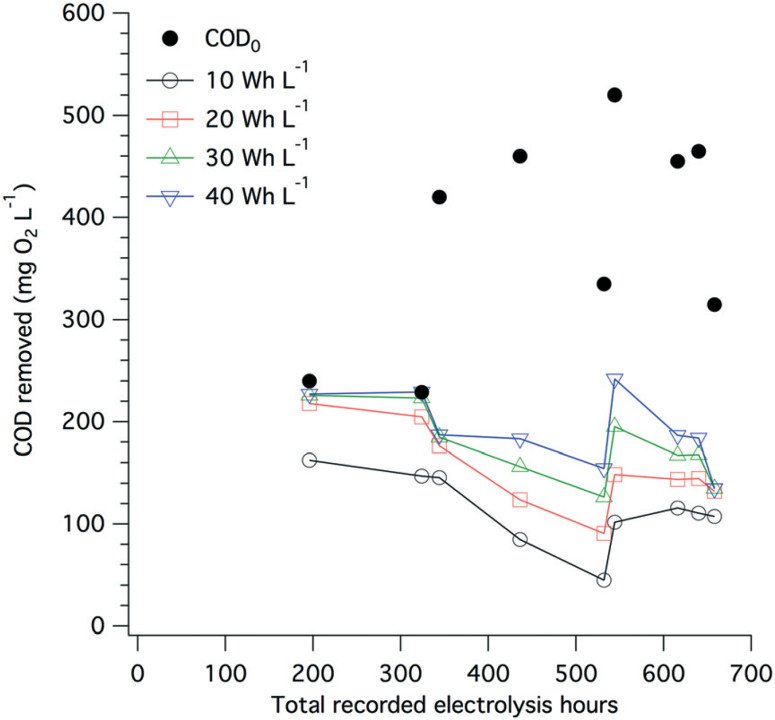
Extrapolated COD removal from toilet wastewater at different levels of electrical energy consumption (10, 20, 30, and 40 W h L^−1^) and over the course of the field testing of the AMD unit. The extrapolation calculation is based on a first-order kinetic model for electrochemical COD removal developed by Martinez-Huitle and Ferro,^51^ see Fig. S7† and Table S1† for curve fittings and calculations. The black dots indicate the COD_0_ value of the wastewater before electrochemical treatment.

The amount of electrical power drawn by the electrode arrays and by the overall system was measured on a regular basis (Fig. S8†). On average, 35 W h were needed to treat 1 L of toilet wastewater, among which more than 95% of the electricity was used by the electrochemical treatment itself and the remaining 5% was used to compensate the power supply losses and to power the pumps. A large share of the energy used during electrolysis is for COD removal, especially when more than 200 mg O_2_ L^−1^ removal is needed (Fig. 8), and the electrolysis energy consumption is between 30 and 40 W h L^−1^. Despite drastic changes in the input COD level and over the course of close to 700 h of toilet wastewater electrolysis, the COD removal energy requirements remained relatively stable at approximately 10 W h L^−1^ for 100 mg O_2_ L^−1^ and up to 40 W h L^−1^ for 200 mg O_2_ L^−1^ initial COD.

## 4.5 Applicability of the technology in the context of a developing country

The AMD prototype unit was connected to a public toilet produced by ERAM Scientific; the “eToilet” had remote monitoring capacity. All of the eToilet uses were recorded over the course of the testing period as well as the number of treatment cycles logged by the AMD unit (Fig. S10†). The treatment capacity of the unit was adequate for the number of users since there was no limitation in the number of eToilet uses from lack of treated water. Issues related to the engineering connections between the eToilet-AMD unit prevented use for more than 6 months. Mechanical and electrical issues detected by the maintenance engineer in residency on the AMD testing site were solved with remote or on-site assistance of the authors. The parts that were replaced during the testing period included pumps that failed for mechanical reasons and failures in the electrical energy storage subsystem (TRUECharge 2 40 A grid to 24 V converter and 12 V Blue Top lead-acid backup batteries, Fig. S3†). The mechanical failures of the pumps were due to fatigue and solids (sand) abrading the impeller and/or the diaphragms. The electrical failures of the energy storage subsystem were probably due to over-drainage events of the batteries when the system was used in the park but disconnected from the grid for very long periods (12 hours or more) and several grid electricity failures. These issues highlight the necessary trade-off between increasing the overall capital expenditure of a system with components prone to less failure such as higher-grade pumps or sufficient solar panels to provide a backup source of power, and managing the operational expenditures due to frequent replacement of parts and grid electricity costs. These issues also highlight the necessity for frequent monitoring of the toilet wastewater treatment system in order to minimize the potential negative health impact on the users. A solution could be in the form of an automatic detection and maintenance system that could investigate the status of the treatment system *via* a suite of sensors and potentially self-repair or provide a step-by-step guide for repairs that necessitate the presence of a technician or a lesser qualified person.

## 4.6 Possible prototype improvements for commercialization

The efficacy of the electrochemical treatment technology to clarify and disinfect toilet wastewater by generating chlorine without addition of water or chemicals makes this technology attractive as a non-sewered sanitation system, especially since it does not depend on the type of toilet used (*e.g*., “westernstyle” flush toilet, squat pan) and does not require specific training or any change of behavior of the user.

Nevertheless, several improvements to the pre-alpha prototypes can be made to increase the robustness and energy efficiency of this electrochemical technology to meet the goals of the RTTC. A replacement of the sedimentation tank at the input of the process (Fig. S1†) by more advanced biological pretreatment technologies such as small-size coupled aerobic/ anaerobic systems^5^ or microbial fuel cells^56^ could effectively decrease the amount of undesired organic and inorganic contaminants entering the electrochemical reactor. This approach would drastically reduce the operational expenses of the system by lowering the amount of electricity needed to complete the electrochemical treatment. Also, the biosolid residuals from the pre-treatment step as well as the filtered materials (Fig. S1†) should be properly decontaminated before being extracted from the system *via* a targeted decontamination process such as ohmic heating.^57^

## 5 Summary

In response to the Bill and Melinda Gates Foundation challenge to “Reinvent the Toilet”, our research group at Caltech developed several self-contained, decentralized waste treatment systems that were designed to treat human domestic toilet waste at its source with discharge to the environment. After toilet flushing the discharged waste is stored in a wastewater tank. After some decantation, the effluent water from the wastewater tank is pumped into an electrochemical reactor array upon demand for the electrochemical oxidation of the residual organic and inorganic constituents. Disinfection is achieved *via in situ* chlorine generation resulting from anodic oxidation of chloride. Electrons released during anodic oxidation flow to the electronically coupled cathodes to produce molecular hydrogen via water reduction. The sequential biological and electrochemical treatment reduces the COD and microbial levels to below WHO agricultural reuse standards, while denitrification takes place due to breakpoint chlorination. In the field-level prototype systems, the treated black water is recycled into flush water reservoirs without significant discharge to the surrounding environment.
