# Supplementary material for: Design and preliminary implementation of onsite electrochemical wastewater treatment and recycling toilets for the developing world
Source: Environ Sci (Camb). 2018 Aug 7;4(10):1439–50. doi: 10.1039/c8ew00209f (PMC7705125; doi:10.1039/c8ew00209f)
Supplement: Supplementary file 1 [file ESWRT-04-1439-s001.pdf]

Electronic Supplementary Material (ESI) for Environmental Science: Water Research & Technology.
This journal is © The Royal Society of Chemistry 2018

Supporting information

Design and preliminary implementation of onsite electrochemical wastewater treatment and recycling toilets for the developing world

Clément A. Cid[1], Yan Qu[1], and Michael R. Hoffmann*[1]

[1]Linde-Robinson Laboratories, California Institute of Technology,
1200 E California Blvd, Pasadena, CA 91125
[2] Trussell Technologies Incorporation,
232 N Lake Ave., Suite 300, Pasadena, California, 91101, USA

Number of pages: 12 (including this cover page)

Number of figures: 8

Number of tables: 1

Supplementary figures

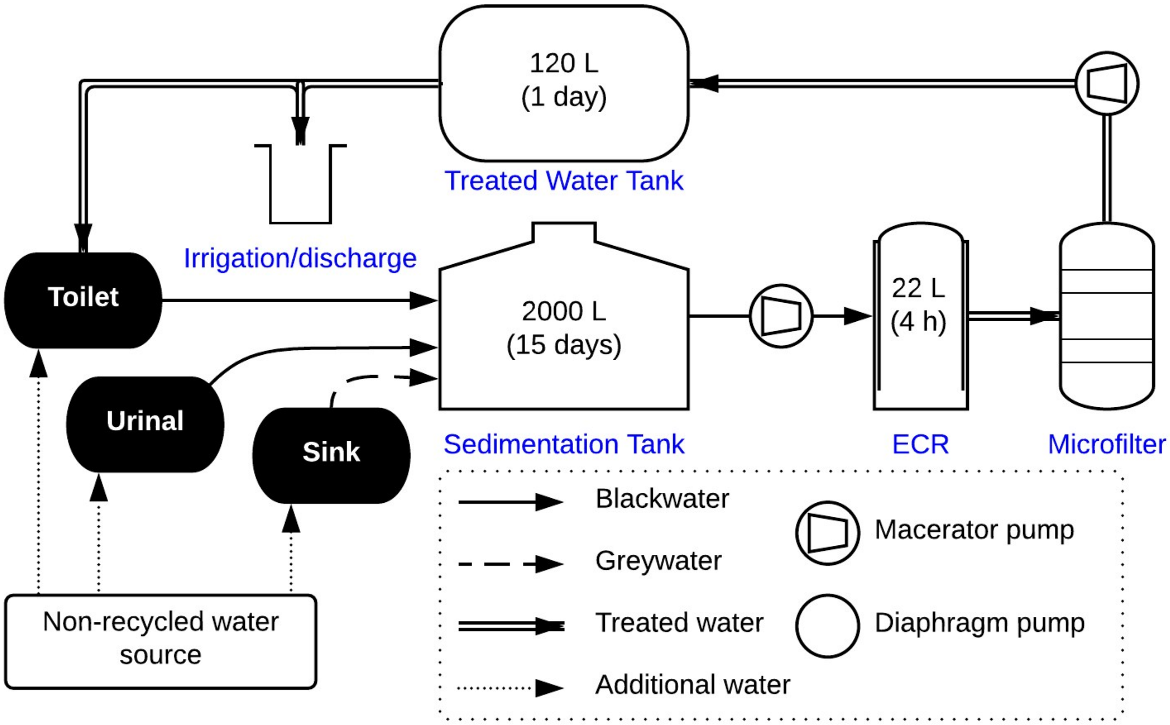

Figure S1: System flow diagram of the self-contained toilet electrochemical treatment system with capacity and residence time of the relevant components. Non-recycled water source could be a connection to a city's water network for personal hygiene and handwashing.

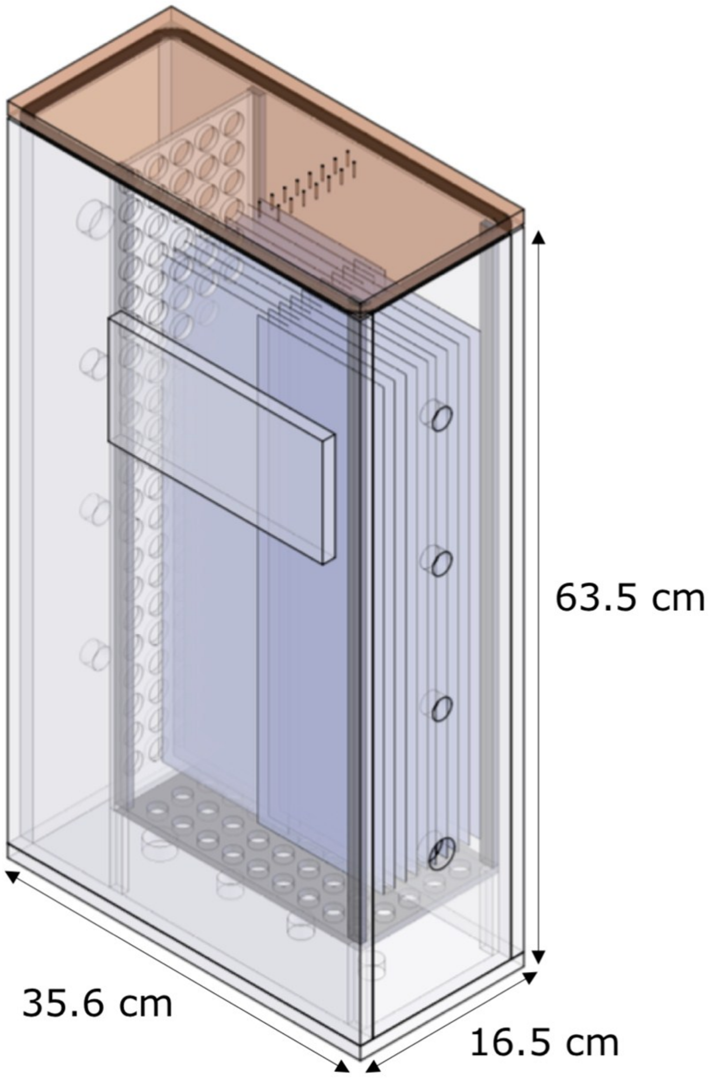

Figure S2: CAD rendering of the electrochemical reactor (ECR) body with an artist view of the electrode array in its core. Dimensions of the outer shell are given as reference.

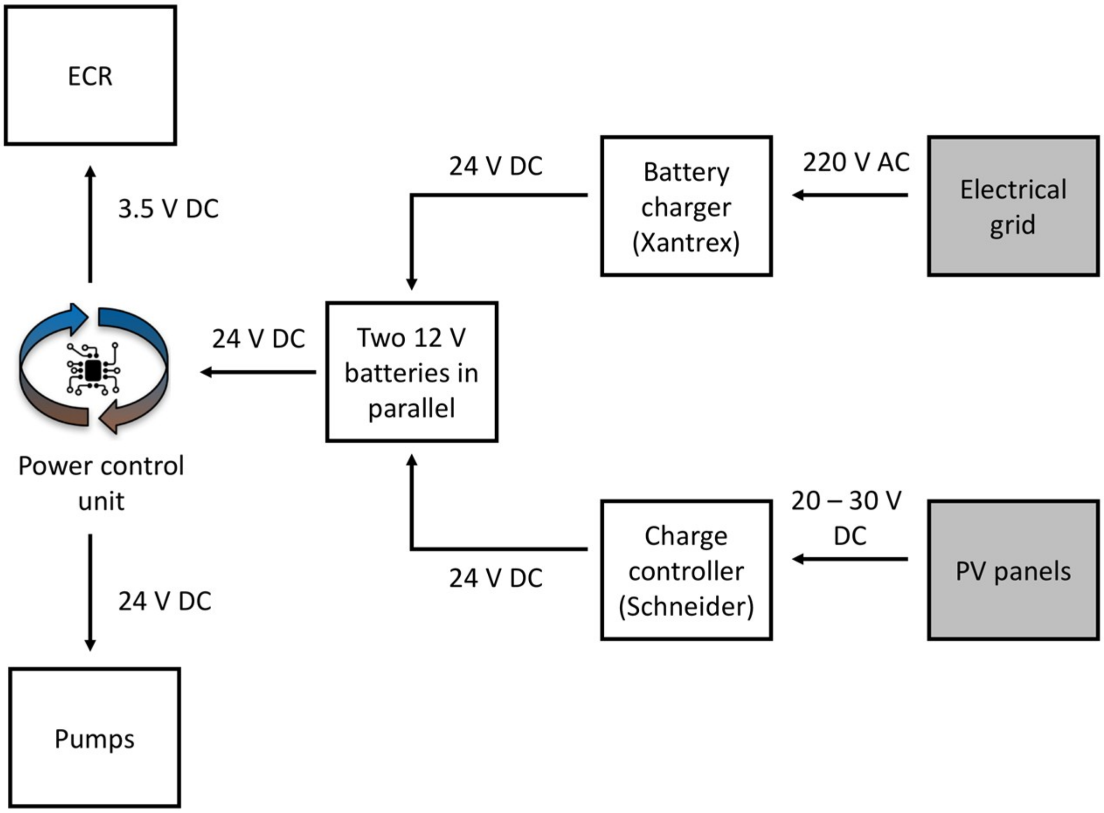

Figure S3: Simplified electrical energy flow diagram of the Caltech Solar Toilet.

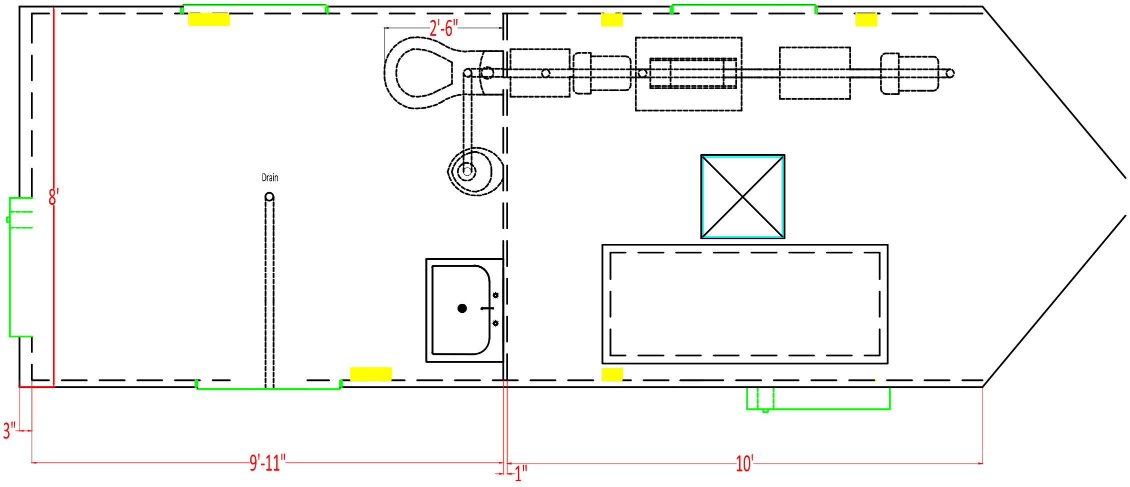

Figure S4: Typical layouts of the self-contained electrochemical treatment systems with a dedicated bathroom located on the left side and a treatment room on the right side.

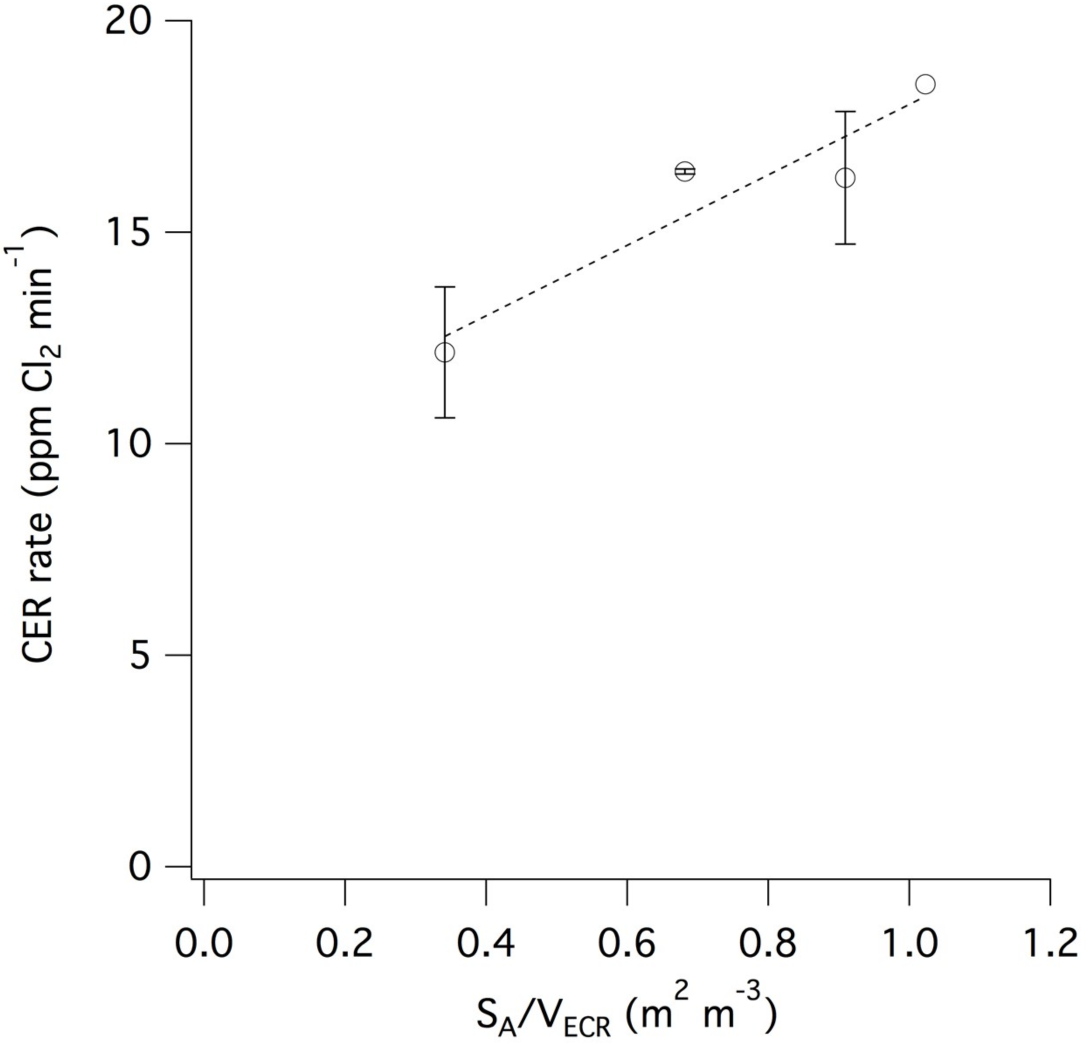

Figure S5: Measured CER rate (ppm Cl_2 min^{-1}) at 3.5 V in 22 L of 20 mM NaCl solution as a function of anodes surface area (S_A, m^2) to reactor active volume (V_{ECR}, m^3). Linear regression equation: CER = 8.3 · (S_A/V_{ECR}) + 9.7 (R^2 = 0.89). Error bars represent ± one standard deviation of 3 replicates except for the data point with highest S_A/V_{ECR}.

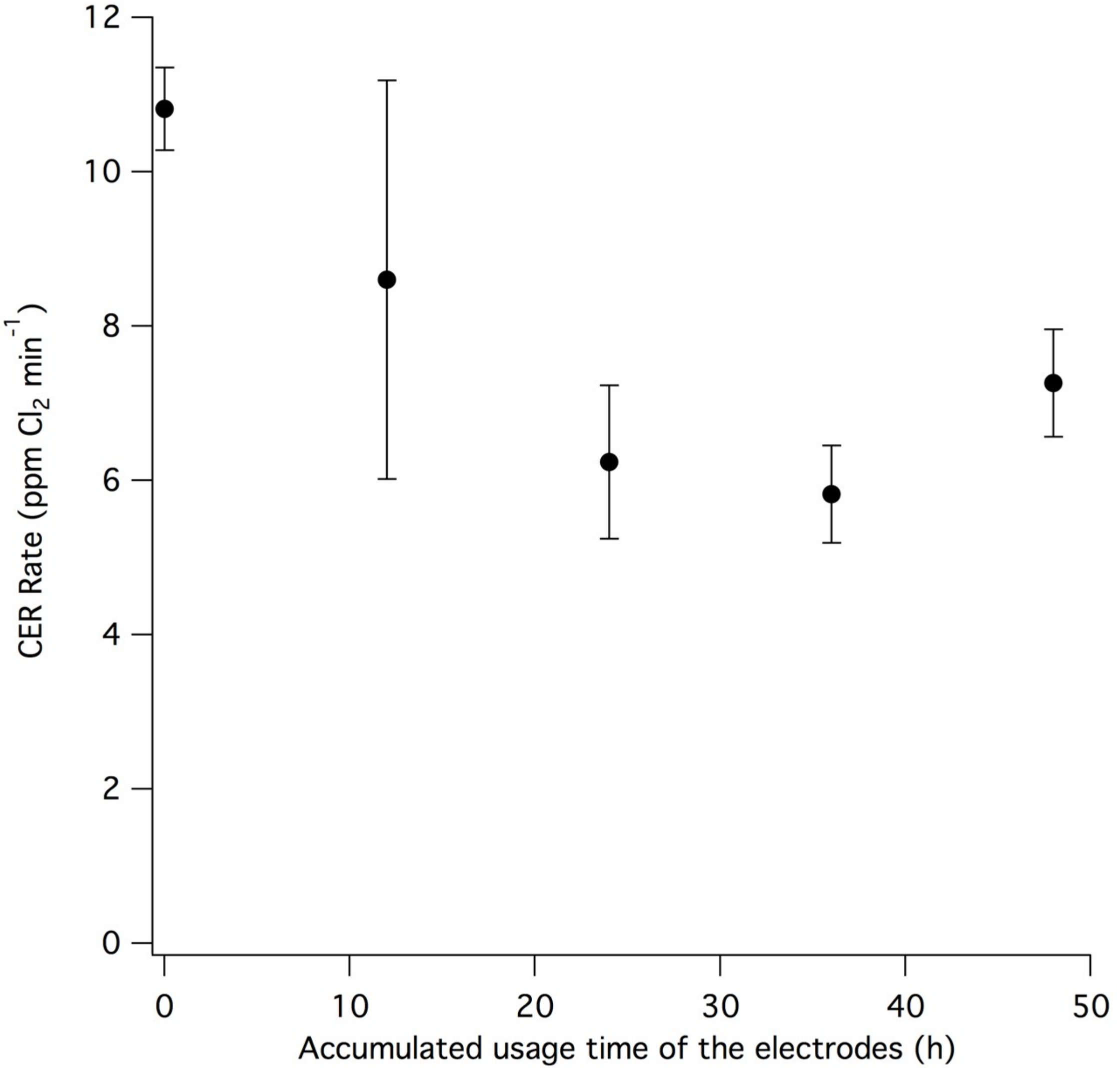

Figure S6: CER rate determined with 20 mM NaCl solution in water after usage of the electrodes for toilet wastewater treatment in PAS unit.

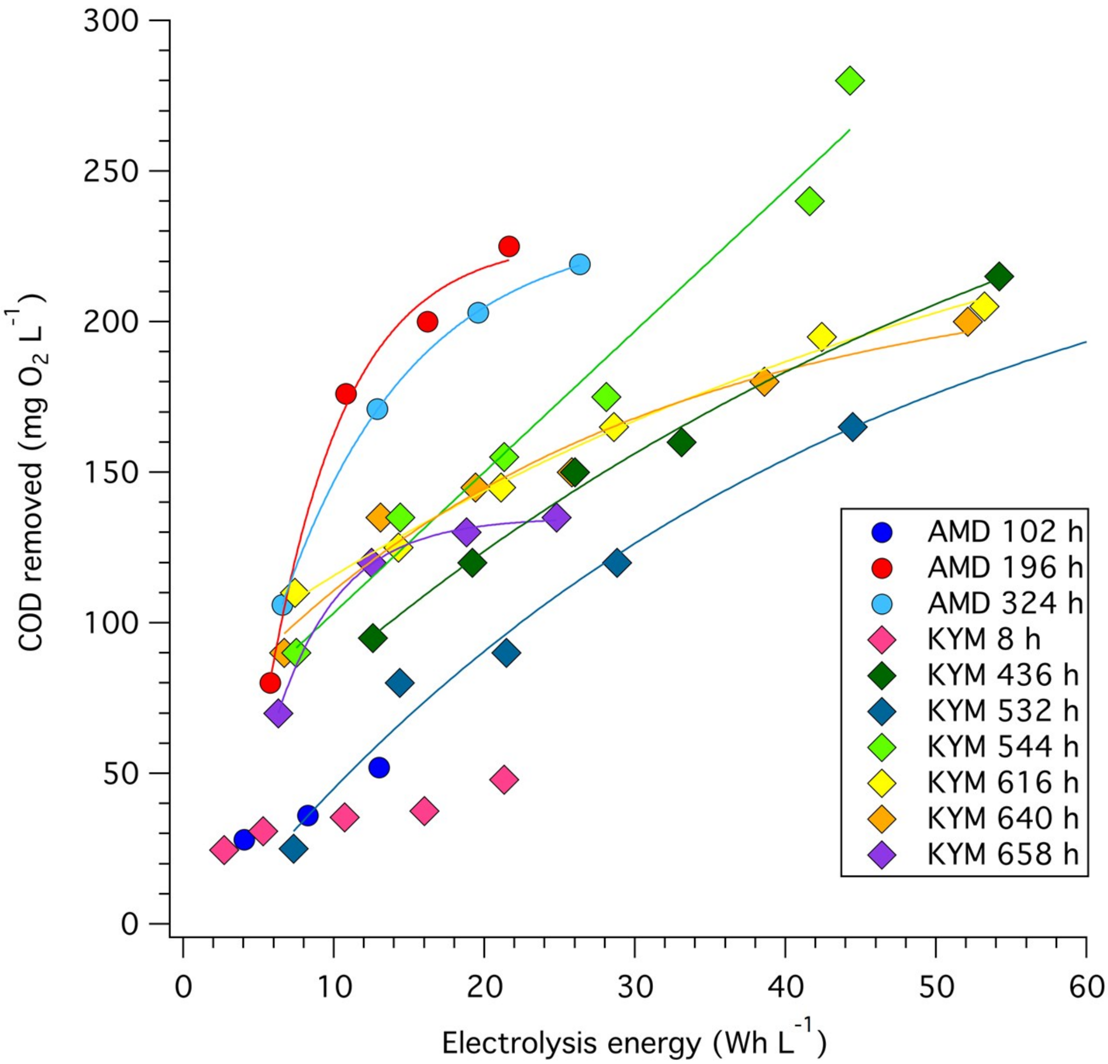

Figure S7: COD removed per Wh L^{-1} during a treatment cycle (4 h to 6 h) after specific acumulated toilet wastewater electrolysis time for prototypes AMD and KYM.

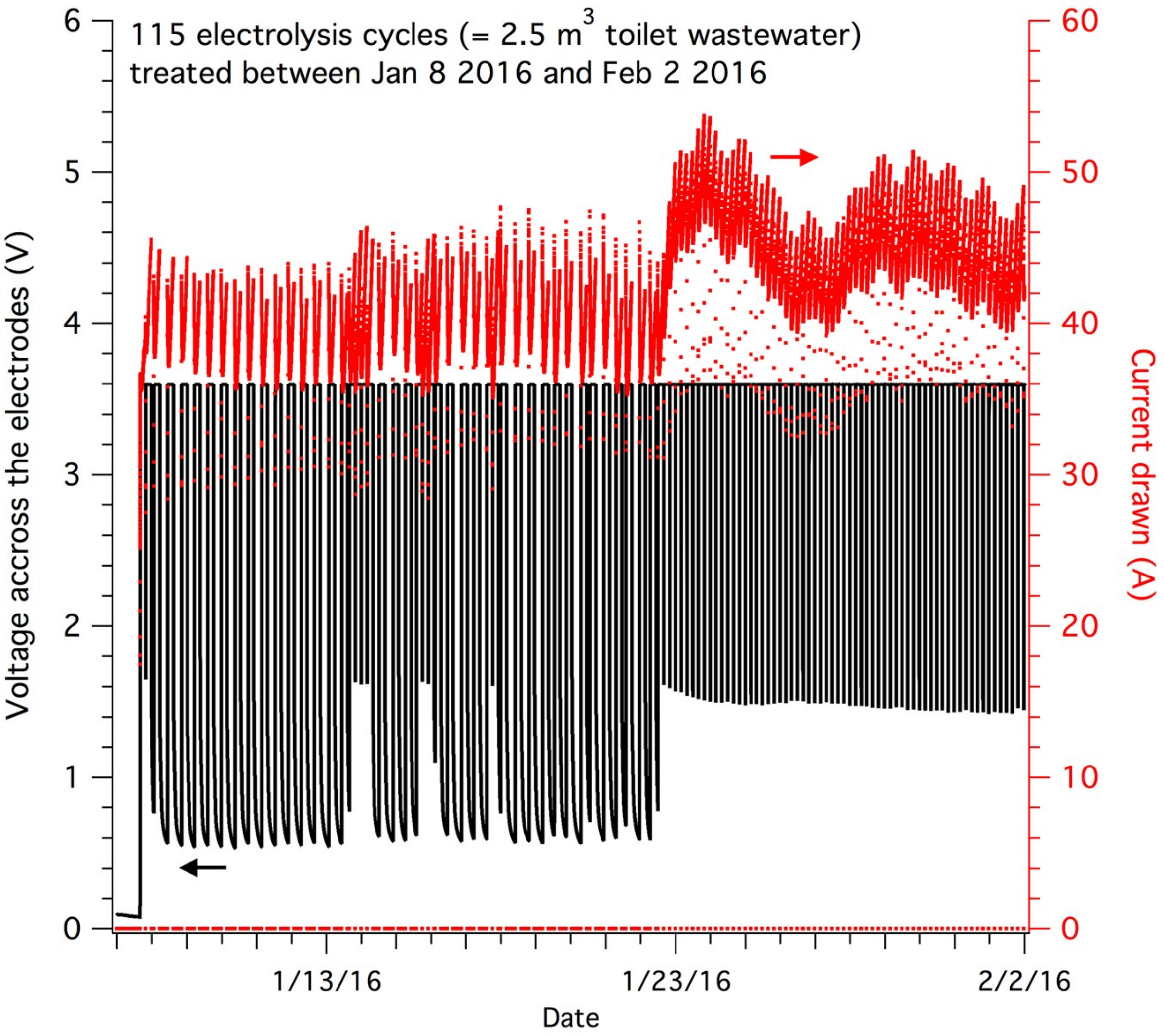

Figure S8: Recorded electrolysis voltage and current of the ECR during a typical month of full usage of AMD prototype. Variations in cycles are due to ECR turning off and on following the automation mechanism Figure 3. The overall increase in current drawn after 01/23/2016 can be attributed to a higher concentration of Cl^- in the wastewater due to recycled treated water being used for flushing. The additional urine of the users of the toilet increases the quantitiy of Cl^- in the system. (see Table 4).

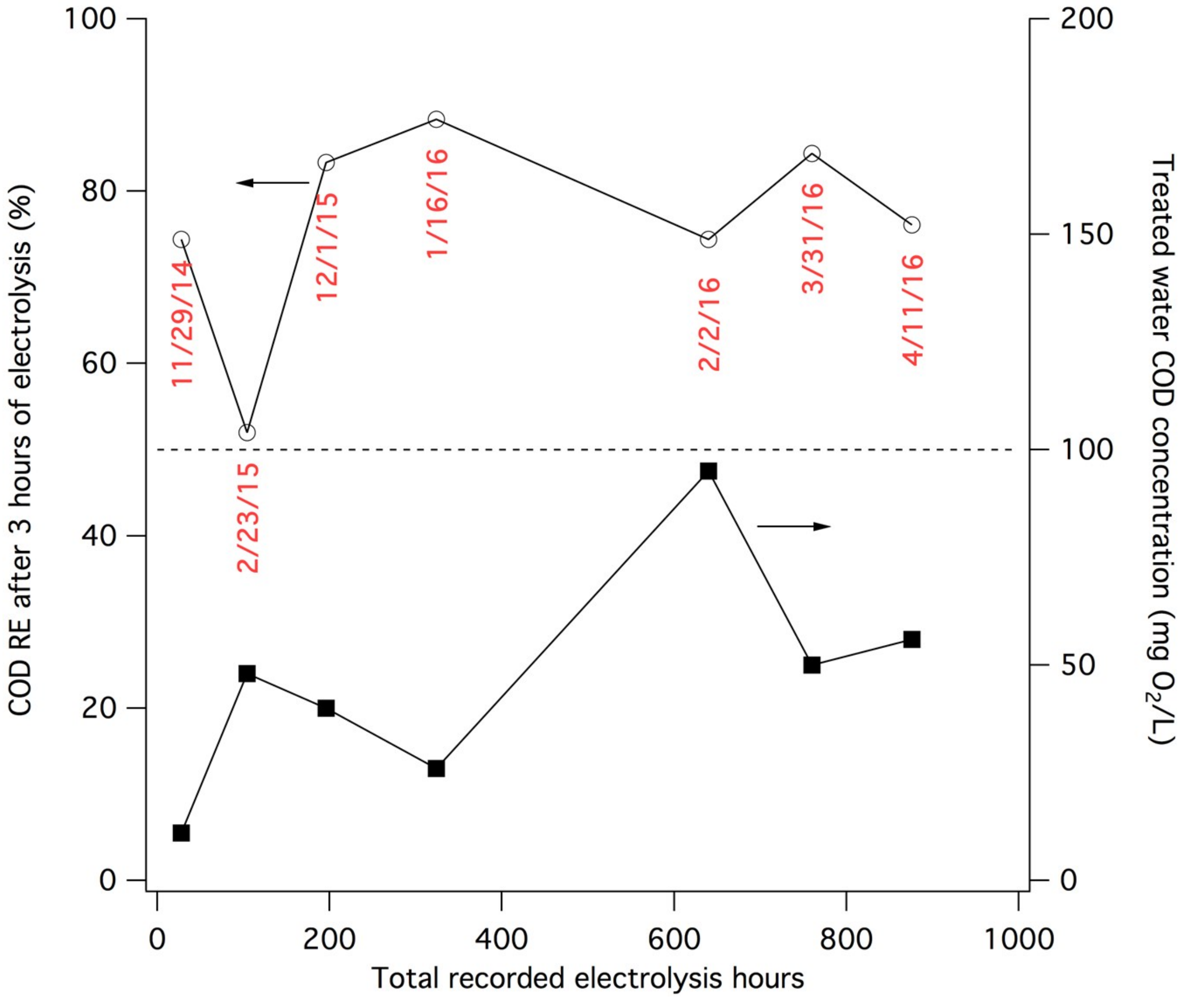

Figure S9: COD removal efficiency (RE) and output COD value of treated toilet wastewater of AMD prototype. Effective sampling dates are written vertically.

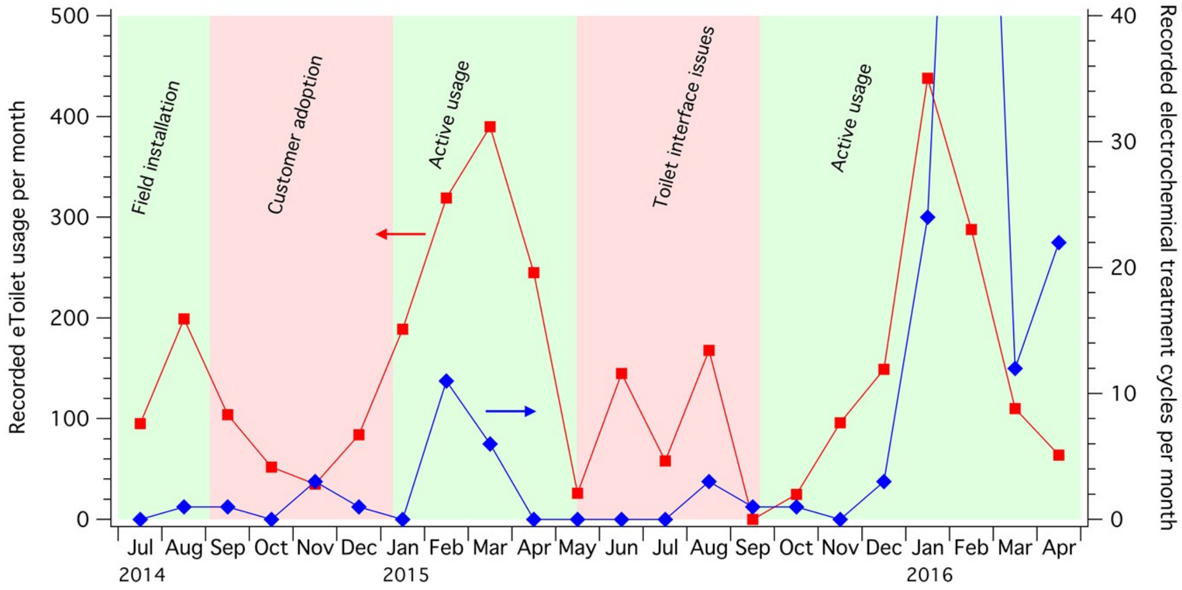

Figure S10: Recorded monthly usage of eToilet (red squares, left) and number of electrolysis treatment cycles per month (blue rhombi, right) during operation of the AMD unit.

Supplementary table

Table S1: Coefficients obtained by computational fit obtained by Igor Pro 6.37 (Wavemetrics) with equation (S1) of the COD removal data measured after the specific acumulated electrolysis times (Figure S7). σ_0, σ_1, and σ_2 correspond to ± one standard deviation of C_0, C_1, and C_2 respectively.

Accumulated Electrolysis Time (h)	C_0	C_1	C_2	A_0	σ_0	σ_1	σ_2
**196**	227	-147	5.16	5.77	14.4	16.5	1.58
**324**	232	-126	8.75	6.56	1.27	1.29	0.22
**344**	188	-78.3	7.82	5.27	-	-	-
**436**	320	-224	55.2	12.6	83.7	81	29.6
**532**	255	-224	40.7	7.33	48.3	44.9	15.6
**544**	3.03E+05	-3.02E+05	6.46E+04	7.50	3.23E+08	3.23E+08	6.9E+07
**616**	286	-178	55.5	7.40	47.5	45.7	21.8
**640**	218	-122	26.4	6.70	30.6	28.2	13.5
**658**	135	-64.9	4.32	6.30	1.79	2.43	0.49

$$COD\ (removed) = C_0 + C_1 exp(-(x - A_0)/A_2) \qquad \text{(S1)}$$
